# Electrically Tunable Defect-Mode Wavelengths in a Liquid-Crystal-in-Cavity Hybrid Structure in the Near-Infrared Range

**DOI:** 10.3390/ma16083229

**Published:** 2023-04-19

**Authors:** Guan-Fu Sung, Shun-Yi Chiu, Yi-Cheng Chang, Yu-Chen Liou, Chin-Pin Yeh, Wei Lee

**Affiliations:** 1 College of Photonics, National Yang Ming Chiao Tung University, Guiren District, Tainan 711010, Taiwan; 2 Institute of Imaging and Biomedical Photonics, College of Photonics, National Yang Ming Chiao Tung University, Guiren District, Tainan 711010, Taiwan; 3 Institute of Lighting and Energy Photonics, College of Photonics, National Yang Ming Chiao Tung University, Guiren District, Tainan 711010, Taiwan; 4 Institute of Photonic system, College of Photonics, National Yang Ming Chiao Tung University, Guiren District, Tainan 711010, Taiwan; 5 Apogee Optocom Co., Ltd., Hsinshi District, Tainan 744094, Taiwan; chinpin@nextapogee.com.tw

**Keywords:** photonic crystals, liquid crystal, photonic bandgap, defect modes, dielectric multilayer, near-infrared spectrometer

## Abstract

This work proposes a novel approach to developing a core component for a near-infrared (NIR) spectrometer with wavelength tunability, which is based on a liquid crystal (LC)-in-cavity structure as a hybrid photonic crystal (PC). By electrically altering the tilt angle of the LC molecules under applied voltage, the proposed PC/LC photonic structure consisting of an LC layer sandwiched between two multilayer films generates transmitted photons at specific wavelengths as defect modes within the photonic bandgap (PBG). The relationship between the number of defect-mode peaks and the cell thickness is investigated using a simulated approach based on the 4 × 4 Berreman numerical method. Furthermore, the defect-mode wavelength shifts driven by various applied voltages are studied experimentally. To minimize the power consumption of the optical module for spectrometric application, cells of different thicknesses are explored for the wavelength-tunability performance of the defect modes scanning through the entire free spectral ranges to the wavelengths of their next higher orders at null voltage. A 7.9 μm thick PC/LC cell is verified to attain the low operating voltage of merely 2.5 V_rms_ required to successfully cover the entire NIR spectral range between 1250 and 1650 nm. The proposed PBG structure is thus an excellent candidate for application in monochromator or spectrometer development.

## 1. Introduction

Photonic crystal (PC) is an intriguing type of optical medium that can be made of periodically arranged dielectric materials, either natural or artificially synthesized. The periodic structure gives rise to a “spatial” feature known as a photonic bandgap (PBG), which impacts the spectral properties of the composite. This fascinating field of research has garnered significant attention since its initial discovery in 1987 [1,2]. Similar to the electronic bandgap found in semiconductors, the PBG structure of PC exhibits a range of wavelengths that are forbidden from light transmission. To intentionally destroy the structural periodicity or introduce disorder into the periodic PC structure, a defect layer can be inserted, resulting in defect modes of appreciable transmittance within the inhibitory region defined by the PBG. The spectral characteristics of these defect modes are determined by the refractive index and thickness of the defect layer [3]. To design tunable resonance wavelengths, many studies have focused on utilizing birefringent liquid crystal (LC) as a defect layer sandwiched between two periodically dielectric multilayers on substrates (known as dielectric mirrors). Over the past half-century, LCs as an anisotropic mesophase in a fluidic state with its constituent molecules oriented in an orderly fashion have gained immense attention in the field of information displays and beyond due to their unique properties of controllable dielectric and optical anisotropy [4]. Apart from their display applications, these properties allow for the manipulation of the refractive index, and hence the resonance wavelengths of the defect modes in such an LC-filled Fabry–Pérot cavity structure [5,6]. As a result, PC/LC cells have shown promise for a variety of applications. In terms of the optical switchable mechanism based on the stimuli-responsive material properties of LCs through the electrically controlled birefringence effect and other field effects, potential applications of LC-induced defect modes in one-dimensional (1D) hybrid PCs have been suggested, including optically pumped lasers [7,8], filters [9,10], optical switches [11,12,13,14], optical communications [15,16], and beyond. In addition to the use of an applied electric field, the tuning of defect modes can be further fulfilled by dominating their transmission through magnetic fields [17,18,19,20], temperatures [21,22,23], and variations in the angle of incidence of light [24,25]. Among the aforementioned tuning methods, the electrical tuning mode is most extensively adopted owing to its ease and effectiveness for implementation. With the emergence of the awareness of global environmental protection, there is a growing need to develop optical devices with low power consumption or those that do not require any electrical stimulus. Consequently, numerous theoretical and experimental studies have been conducted to demonstrate optically stable defect modes (at null voltage), with the aim of reducing the power consumption. Examples of such devices include bistable photonic devices [26,27], tristable photonic devices [12,28], and low-voltage-consumption photonic devices [29,30], which have potential applications in commercial products of various markets aligned with a green future.

To date, spectroscopy has been ubiquitously used in academia and industry on account of its ability to reveal the interactive relationship between objects and electromagnetic radiation through the measurement of their electromagnetic or spectral properties. This has led to a revolution in a broad range of applications, including sensing [31,32,33], imaging [34,35,36], communications [37,38,39], and so on beyond fundamental sciences. However, due to the intrinsically bulky size of traditional spectrometers and the limited output power of their light sources, they are often not suitable for portable or versatile use in a wide range of scenarios [40]. Fortunately, commercial palm-sized, fiber-optic spectrometers have entered the market, providing convenient and compact analytical tools since the 1990s, such as an Ocean Optics S1000 spectrometer inducted in 1992 [41] and its successor models. Recent advancements in the utilization of LC-in-cavity structures characterized by wavelength tunability of defect modes can be potentially applied to the alternative development of small-volume spectrometers featuring simultaneous scanning in multichannel. This concept offers great potential for portable and convenient use in a good variety of domains, addressing the inherent limitations of traditional monochromators or spectrometers. Although spectrometers with small sizes have been manufactured for more than three decades, spectrometers of this kind highlighted by the working principle of tunable defect modes in a PC/LC with a wavelength scanning function, especially in the near-infrared (NIR) wavelength (*λ*) range have been scarcely discussed. Though it is known that doped nanoparticles in an LC material system may reduce power consumption [42], there are some potential issues associated with the additives in LC mixtures, such as aggregation, instability, unreliability, complex processing, and higher cost. Therefore, an alternative approach that can reduce power consumption without the need for adding nanoparticle material is desirable.

In this study, we propose a low-power-consumption optical module for a miniature NIR spectrometer based on the spectral properties of defect modes within a PBG structure as a hybrid PC. The NIR wavelengths are generally defined as the broad range of electromagnetic radiation that extends from approximately 780 nm to 2500 nm. Here, we are more interested in a narrower range (*λ* = 1250–1650 nm) in that most high-quality optical fibers (used in communications) operate within this wavelength range. Accordingly, our design is tailored for the interested NIR spectrum (*λ* = 1250–1650 nm), achieved by sandwiching nematic LC material between two dielectric multilayers. To investigate the correlation between the defect-mode wavelengths and cell thickness (*d*) in the NIR spectrum, we measured the refractive indices of the eutectic nematic LC E7 at five wavelengths by using an Abbe refractometer and deduced the extended dispersions at *λ* = 1250–1650 nm. This permitted us to input the appropriate birefringence parameter into simulation software, DIMOS LCD (1D), to calculate the optimized number of defect modes and simulate the transmission spectrum of the hybrid PC/LC cell using the 4 × 4 Berreman matrix. In spite of the fact that the Berreman method for numerical calculations is employed in the algorithms of commercial software, other useful methods, such as the finite-difference time-domain method, can sometimes be preferable for computational studies of propagations of light through LC molecules [43]. We fabricated cells of different thicknesses to examine the separation between two adjacent defect modes and the blueshift variations in each defect-mode peak with various voltages with the aim to reduce electric consumption. In order to effectively reduce power consumption, we proposed a novel approach that involves intentionally increasing the number of defect-mode peaks in a PC/LC hybrid photonic structure. This is achieved through the increase in cell thickness, which results in a reduced necessary shift spam of a defect-mode peak. On the basis of our experimental and simulation findings, we selected a hybrid PC/LC cell with a cell gap of 7.87 μm and successfully manifested the feasibility of concurrently segmental scanning over the 400 nm wide spectrum by using externally applied voltage as low as 2.5 V_rms_. While previous studies have intensively explored spectral properties of photonic PC/LC structures [44], there is currently a lack of detailed study of electrooptic performance of defect modes for applications in wavelength-tunable multichannel filters or spectrometric instruments underlain by simultaneous multichannel wavelength scanning. The proposed design addresses this gap and offers a promising technique for an alternative type of miniature spectrometer in the NIR regime. The LC-in-cavity structure composed of two dielectric mirrors can be further utilized to extensively develop novel optical devices.

## 2. Materials and Methods

### 2.1. Materials

**Figure 1:**
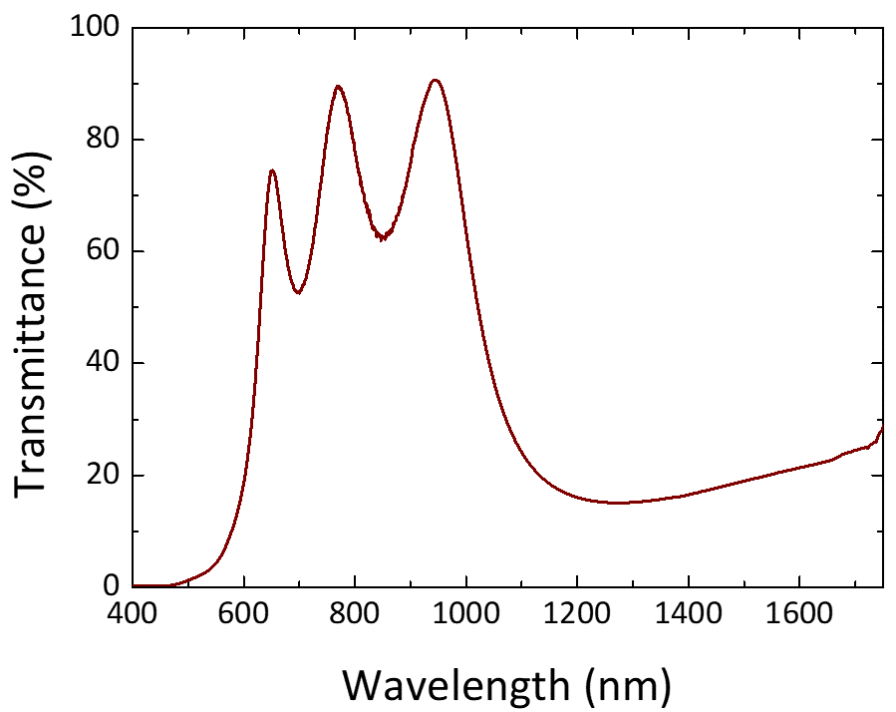
Transmission spectrum acquired from a glass substrate coated with a dielectric multilayer and an ITO film. A Shimadzu UV-2600 UV-Vis spectrophotometer and an Avantes AvaSpec-NIR256-1.7-EVO spectrometer were employed in tandem to produce the spectrum spanning from 400 to 1750 nm.

The nematic LC E7 (Merck, New York, NY, USA) used in this study has positive dielectric anisotropy Δ*ε* = 14.3 (measured at the frequency *f* of 1 kHz and temperature *T* of 20 °C). It also exhibits an extraordinary index of refraction *n*_e_ = 1.7472, ordinary index of refraction *n*_o_ = 1.5217, and birefringence Δ*n* ≡ *n*_e_ − *n*_o_ = 0.2255 (measured at *λ* = 589 nm and *T* = 20 °C). To induce defect modes within a given PBG in the NIR range, we used multilayer films fabricated by Apogee Optocom Co., Ltd., each of which consists of four alternating dielectric layers of a high-refractive-index (*n*) material (a-Si:H, *n*_H_ = 3.51016 at *λ* = 1508.9 nm, and thickness *d*_H_ = 107.47 nm) and a low-refractive-index substance (SiO_2_, *n*_L_ = 1.45629 at *λ* = 1508.9 nm and *d*_L_ = 259.03 nm) deposited on a 1.1 mm thick glass substrate to form a dielectric mirror. An indium–tin-oxide (ITO) electrode was then coated atop each multilayer. The ITO film has a thickness of 191.53 nm and a refractive index of 1.96956 + 0.001*i* at *λ* = 1508.9 nm. Figure 1 portrays the transmission profile of such a glass-multilayer-ITO substrate used for constructing a PC/LC cell to be described in Section 2.2.

### 2.2. Sample Preparations

**Figure 2:**
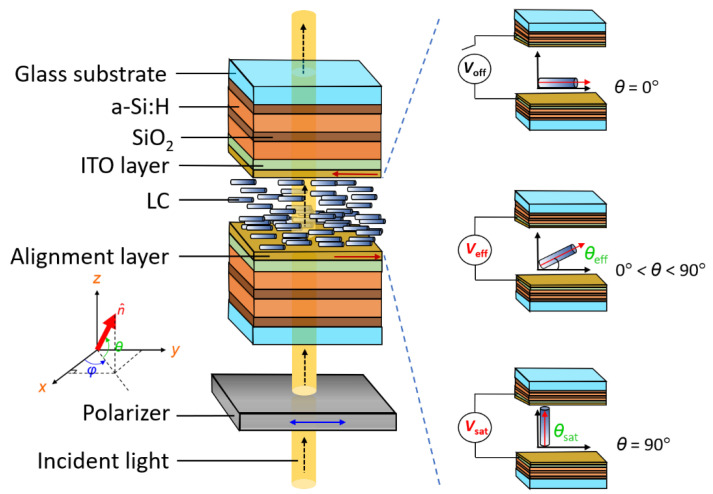
Schematic of the symmetrical configuration of the 1D PC/LC cell used to generate electrically tunable defect modes in the PBG specified by the dielectric multilayer. Note that the polarization angle (between the polarization direction of the linearly polarized beam and the unperturbed optic axis of the LC) is fixed at 0°.

Each of the conductive glass substrates was coated with a homogeneous alignment layer of polyimide (SE-150, Nissan Chemical, Tokyo, Japan) using a spin-coating process, followed by baking and mechanical rubbings in anti-parallel directions of two paired substrates to ensure planar alignment of the LC molecules. Empty cells were fabricated by assembling pairs of the thus treated substrates separated by ball spacers. E7 was heated to isotropic phase (at 100 °C) and then infiltrated into the empty cells by capillary action. This means that the LC-in-cavity structure under investigation was prepared by sandwiching a nematic LC between two identical multilayer films, as schematically shown in Figure 2. A high-contrast NIR polarizer (#36-654, Edmund Optics, Barrington, NJ, USA) with a functional wavelength range from 650 to 1700 nm was placed between a PC/LC cell and the light source. Applying voltage across the cell gap beyond a threshold can cause a change in orientation of the LC director (defined as the local average axis of the long molecular axis), leading to a variation in the refractive index. From the index ellipsoid, the effective refractive index *n*_eff_ (*θ*) of the (uniaxial positive) birefringent nematic LC can be calculated as follows [45]:

(1) $$n_{o}\leq n_{\mathrm{eff}}(\theta )=\frac{n_{o}n_{e}}{\sqrt{\left[n_{e}^{2}\sin^{2}\theta +n_{o}^{2}\cos^{2}\theta \right]}}\leq n_{e}$$

where *n*_o_ and *n*_e_ are the ordinary and extraordinary refractive indices, respectively, and *θ* is the angle between the optic axis (i.e., the nematic director) and the polarization direction of a linearly polarized beam. With a fixed angle of incidence such as the case of normal incidence as adopted in this work throughout, *n*_eff_ is simply determined by the LC director whose orientation can be readily tuned by externally applied voltage *V*. The electrical response originating from dielectric anisotropy in the LC bulk allows for effective manipulation of *n*_eff_ (*θ*) under various applied voltages, inferring that *n*_eff_ is a function of the externally applied electric field. The working of this concept is schematically presented in Figure 2. In the absence of an electric field (*V*_off_), *n*_eff_ = *n*_e_ because the molecular axis of LC in the unperturbed planar state is oriented parallel to the polarization direction along the *y*-axis of the linearly polarized beam. This condition will bring about all defect modes associated with only *e*-waves. At an effective voltage (*V*_eff_ beyond the Fréedericksz transition voltage) causing the dielectric response to overcome the influence of the boundaries, *n*_eff_ decreases and all defect modes blueshift with increasing voltage, depending on the *V*-controlled angle *θ*_eff_. As the applied voltage reaches the saturation voltage (*V*_sat_), the LC director orients itself perpendicularly to the substrate plane, resulting in a homeotropic configuration and, in turn, the minimized effective refractive index *n*_eff_ (*θ*_sat_) = *n*_o_, where *θ*_sat_ approaches 90°. This is a limiting case in which all the defect-mode peaks in the transmission spectrum reduce to the *o*-mode signals.

### 2.3. Simulation and Measurements

**Figure 3:**
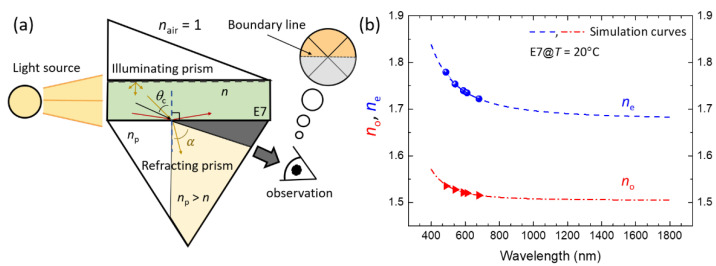
(**a**) Schematic of the working principle of the Abbe refractometer and (**b**) wavelength-dependent refractive indices of E7 at *T* = 20 °C. The symbol *n*_p_ denotes the refractive index of the prism and *θ*_c_ is the critical angle. The simulated curves extended to the NIR region are the dispersion functions obtained by fitting the experimental data at five visible wavelengths into the extended Cauchy equation [50].

Using simulation software DIMOS LCD (1D) 2.0 version, we performed a simulation approach based on the 4 × 4 Berreman numerical method prior to the transmission measurement. To grasp the refractive parameters required for the simulation of transmittance of the defect modes from the LC-in-cavity structure (viz. E7-filled Fabry–Pérot cavity) within the PBG in the NIR spectrum, we exploited an Abbe refractometer (ATAGO DR-M4, Tokyo, Japan) with five dissimilar filters (at *λ* = 486 nm, 540 nm, 589 nm, 610 nm, and 680 nm) to extrapolate the NIR wavelength-dependent refractive indices *n*_e_ and *n*_o_ of E7. Figure 3a illustrates the measurement mechanism of the refractometer used. Laboratory NIR transmission spectra were acquired with a fiber-optic spectrometer (Avantes AvaSpec-NIR256-1.7-EVO, Apeldoorn, The Netherlands) along with a halogen light source (AvaLight-HAL-S-Mini tungsten, Apeldoorn, The Netherlands). PC/LC cells were either unperturbed (*V* = 0) or driven by voltage fixed at *f* = 1 kHz supplied from a function generator (Tektronix AFG-3022B, Beaverton, OR, USA) in connection with a power amplifier (TREK Model 603, New York, NY, USA) with 50× magnification. All measurements were taken at room temperature (*T* = 20 °C).

## 3. Results and Discussion

### 3.1. Birefringence in the LC Material E7

Figure 3b depicts the measured refractive indices of E7 at five different wavelengths in the visible spectrum and their fitting curves extended to the NIR region following the extended Cauchy equation. The experimental results indicate that both refractive indices of E7 decrease as the wavelength increases. The Cauchy equation is known to be of use for finding the refractive index of an isotropic gas or a liquid compound as transparent fluidic material [46]. This equation can also be applied to birefringent materials such as LC [47]. To enable simulation of defect-mode transmittance of the hybrid photonic structure in the NIR wavelength range, we adopted the extended Cauchy equation to extrapolate the refractive indices in the NIR range by fitting the experimental data (represented by the symbols in Figure 3b). According to the Cauchy model, the refractive indices can be expressed as [48]:

(2) $$n_{e,o}\left(\lambda \right)=A_{e,o}+\frac{B_{e,o}}{\lambda^{2}}+\frac{C_{e,o}}{\lambda^{4}}$$

where the Cauchy coefficients *A*_e,o_, *B*_e,o_, and *C*_e,o_ can be determined by fitting Equation (2) into the refractive indices measured. Deduced from Figure 3b, the refractive indices of E7 in the NIR range of interest (namely, 1250–1650 nm) are *n*_e_ = 1.6877 ± 0.0012, *n*_o_ = 1.5061 ± 0.0001, and Δ*n* = 0.1816 ± 0.0012, exhibiting negligible variation. Although we were unable to directly measure refractive indices at NIR wavelengths due to instrumental limitations, the simulation curves calculated by the extended Cauchy equation provide reliable values of refractive indices in the NIR wavelength range. Indeed, we referred to Tkachenko and coworkers’ experimental study presenting measured optical dispersion curves in the wavelength range of 0.5 to 1.7 μm and are confident that our deduced data at NIR wavelengths are in good agreement and thus undoubtedly acceptable [49].

### 3.2. Simulation of the Transmission Spectra of PC/LC Structures

**Figure 4:**
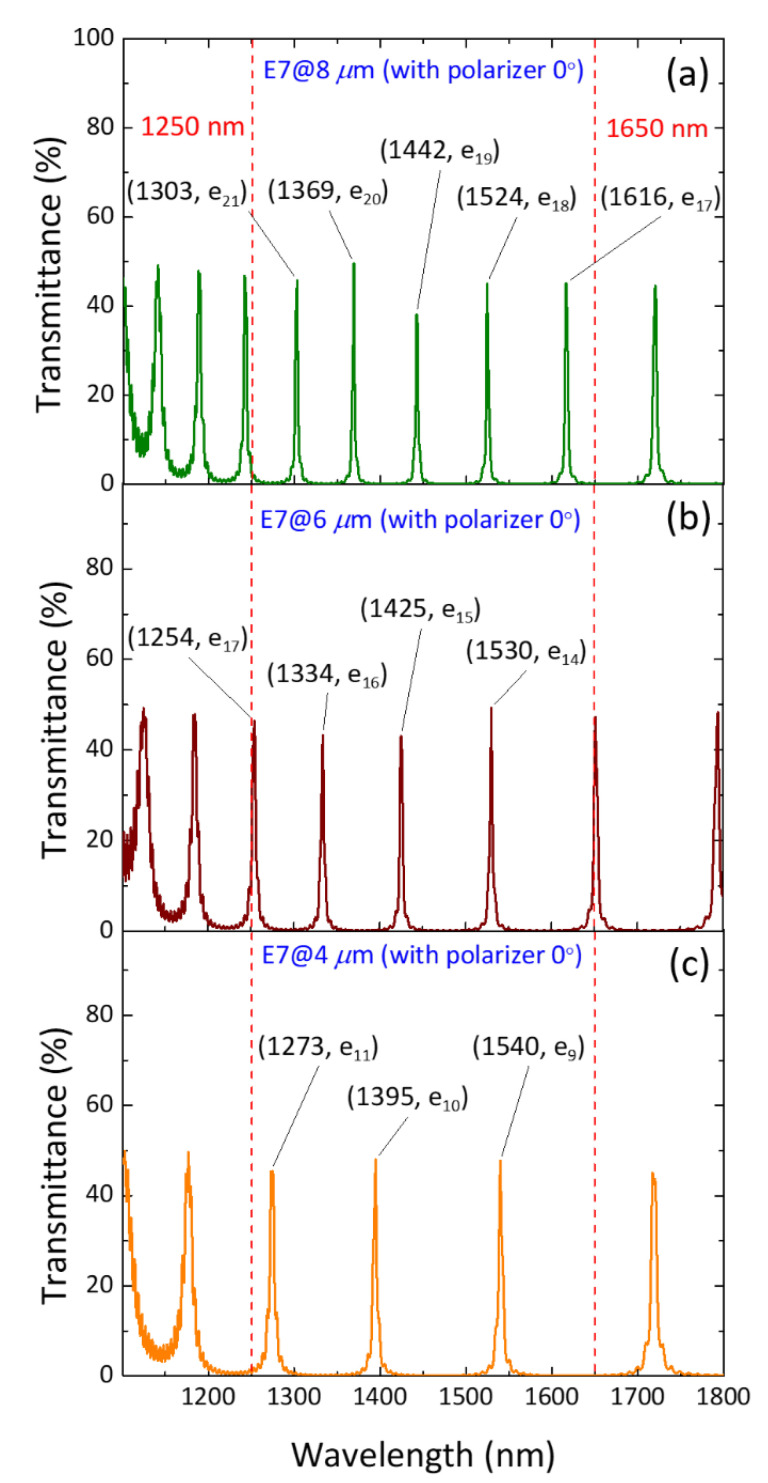
Simulated polarization transmission spectra of three PC/LC cells for a normally incident beam of linearly polarized light at zero voltage. The cell thicknesses considered are (**a**) 8 μm, (**b**) 6 μm, and (**c**) 4 μm. The four-digit number in a pair of parentheses specifies the wavelength in nm and the subscript *m* in a label e*_m_* stands for the order of an *e*-wave resonance in the microcavity.

Figure 4 illustrates the simulated transmission spectra of three homogeneously aligned PC/LC cells of 8 μm, 6 μm, and 4 μm in thickness from which linearly polarized light emerges in the field-off (*V* = 0) condition, presenting all *e*-mode spectral peaks because of the polarization angle of 0° of the incident beam. Note that the polyimide alignment layers are not taken into consideration in the simulation. In order to develop an electrically tunable component in conjunction with, say, a Z-block prism, for spectrometric application, the number of defect modes at specific wavelengths was carefully examined to ensure the transmission peaks spanning the entire selected wavelength range of 1250–1650 nm in the course of the ramp-up of applied voltage. The wavelengths of the transmission maxima, viz. the defect-mode peaks, can be determined by the following equation [17]:

(3) $$\lambda_{e}=\frac{2dn_{e}}{m_{e}}$$

where *m*_e_ is the serial number of defect modes for *e*-waves and *d* is the thickness of the LC defect layer. As depicted in Figure 4a, a cell with a thickness of 8 μm yields 5 defect-mode signals, corresponding to the *e*-mode orders from 21 to 17, in the wavelength range of 1250–1650 nm. For the case of *d* = 6 μm as shown in Figure 4b, four defect modes, assigned to the order numbers from 17 to 14, are observed. Figure 4c shows the polarization spectrum of the PC/LC microcavity of a further reduced thickness of *d* = 4 μm. It features 3 defect-mode peaks, namely, e_11_ (at 1273 nm), e_10_ (at 1395 nm), and e_9_ (at 1540 nm). It is obvious from Figure 4 that the number of defect modes reduces and the free spectral range defined as the wavelength separation between two neighboring defect modes increases as the cell thickness decreases. Spectral results attained in additional cell-gap conditions are jointly presented in Table 1. The table displays the wavelengths of the simulated defect modes and their corresponding *e*-mode order numbers in the NIR spectral range of interest, where a wavelength is followed by the order number in square parentheses. Here, the particular consideration of *d* = 4.6 μm for simulation stemmed from the availability of spacers in the laboratory.

### 3.3. Electro-Optical Response of the PC/LC Structures

Electrical tunability in the wavelength of the defect modes was studied experimentally. To diminish operating voltage, our intuition was to decrease *d* to consecutively increase the equivalent electric field that is inversely proportional to the cell gap.

**Figure 5:**
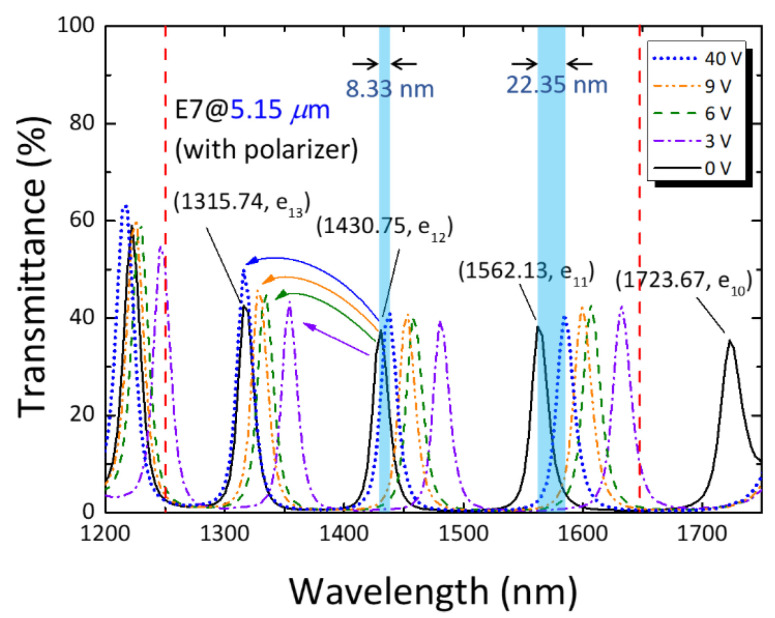
Polarized transmission spectra of the 1D PC/LC cell of *d* = 5.15 μm at various voltages.

The experimental NIR spectra of a 5.15 μm thick PC/LC cell driven by various voltages are shown in Figure 5. The transmission peaks correspond to the one-by-one defect modes, whose *e*-mode order numbers are designated by Equation (3). Although 4.6 μm spacers were used to fabricate the microcavity, the actual cell thickness of the handmade product is 5.15 μm. Figure 5 unambiguously delineates three distinct *e*-mode peaks identified as e_11_ (*λ* = 1562.13 nm), e_12_ (*λ* = 1430.75 nm), and e_13_ (*λ* = 1315.74 nm) within the wavelength range of 1250–1650 nm. As mentioned previously, the effective refractive index of the birefringent defect layer is governed by the applied electric field. Here, one can see a blueshift of each spectral feature (i.e., defect-mode peak) upon applying an AC voltage. The continuous shift in defect-mode wavelength induced by increasing voltage empowers scanning in the interested NIR region. The wavelength of a defect mode decreased with increasing voltage. As *V* increased from 0 to *3* V_rms_, significant reorientation of the LC molecules occurred, engendering a remarkable increase in tilt angle and, in turn, a drastic reduction in the effective refractive index. As the applied voltage was raised to *V* = 40 V_rms_ to severely arouse the LC molecules to render a tilt angle from *θ*~0° to *θ*~90° (Figure 2), all defect-mode signals reached saturated shifts to the shortest possible wavelengths near those of their adjacent higher orders in the field-off condition. In other words, an *o*-mode—as a limiting case of its *e*-mode at significantly high voltage—was in the vicinity of the *e*-mode wavelength of the next higher order (at a shorter wavelength). Noticeably, at such a high voltage, an *e*-mode of a lower order may not be able to move to the shorter wavelength of the next higher *e*-mode order at *V* = 0 V although the shift of a lower-order defect mode was larger. Figure 5 evidently shows that, even at the maximal voltage of *V* = 40 V_rms_ employed in this work, two defect-mode (e_10_ and e_11_) peaks could not blueshift across the spectrum to the next order of the unperturbed defect modes, leaving a wavelength gap of ~22 nm between the defect-mode peak of o_10_ (i.e., e_10_ at *V* = 40 V_rms_) and the e_11_ defect-mode peak at *V* = 0, and a wavelength gap of ~8 nm between the peak of o_11_ (i.e., e_11_ at *V* = 40 V_rms_) and the e_12_ spectral peak at *V* = 0.

Deduced from the experimental data, Table 2 displays the overall electrically tunable property of the defect-mode wavelength by varying external voltage, as demonstrated by the distinct degrees of blueshift. Taking the e_12_ peak as an exemplary representative, the table reveals that the defect mode shifted by approximately 48 nm (77 nm) at *V* = 2 V_rms_ (3 V_rms_), whereas the shift was ca. 95 nm at *V* = 6 V_rms_. The most dramatic change in voltage-dependent wavelength of a defect mode occurred between 2 V_rms_ and 3 V_rms_. This finding can be more vividly observed by plotting a *λ* (*V*) curve or the first derivative of the wavelength with respect to voltage (d*λ*/d*V*) as a function of the voltage. The *V*-dependent nature is attributable to the variation in LC director under the influence of the electric field [26]. As *V* increased further, the tilt angle was progressively enlarged, eventually leading to a limited variation in *n*_eff_ approaching *n*_o_ (in accordance with Equation (1)) and to a saturated blueshift.

**Figure 6:**
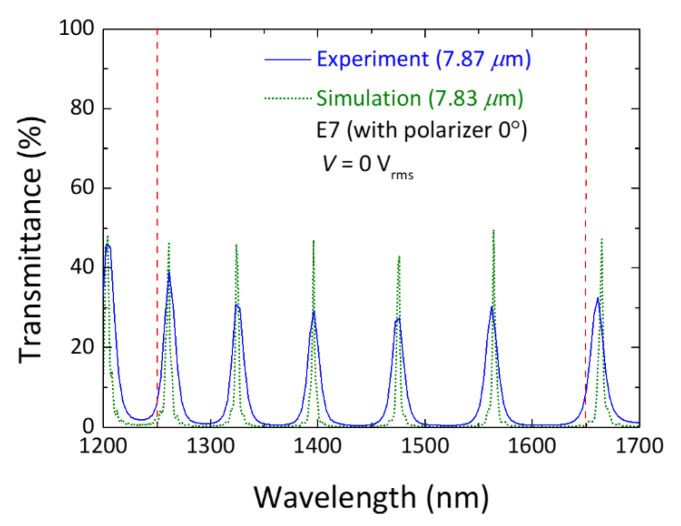
Experimental (blue solid curve) and simulated (green dotted line) polarized transmission spectra of the PC/LC microcavity with a large cell gap determined by 8 μm spacers at zero voltage. The spectral peaks specify the wavelengths of *e*-mode signals of successive orders of interference.

Because of the limit that makes the *V*-induced blueshift of a defect mode unreachable to the desired shorter wavelength designated by its higher-order defect mode at zero voltage, our proposed approach may seem to have a potential weakness for application to atypical scanning spectrometers. To ensure that the wavelength range of interest can be fully scanned by moving defect-mode channels of the PC/LC cell subject to driving voltage, increasing the number of defect modes in the PBG by increasing *d*, as confirmed experimentally or through spectral simulation, may offer a solution in that it will decrease the required maximal range conditioned by the free spectral range for each defect-mode movement. We thus carried out experimental and simulation analyses, targeting an LC-filled Fabry–Pérot cavity of a thicker gap (*d* = 7.87 μm). Figure 6 shows five transmission peaks produced in the microcavity at *λ* = 1250–1650 nm, which agrees well with the simulated spectrum. (Here the simulation was adjusted to a spacer thickness of *d* = 7.83 μm to better resemble the experimental results.) Of particular interest is that our statistical analysis revealed the uncertainty in the calculated cell thickness to be within ±0.05 nm in our experimental results. This justified our adoption of the cell thickness of 7.83 μm in simulation to better match the defect-mode spectral properties. It may indicate that the actual gap of the fabricated PC/LC cell is closer to 7.83 μm instead of 7.87 μm. The experimental transmission of the defect-mode peaks was lower than the simulated one, presumably due to imperfect parallelism of the dielectric mirrors as well as spectral absorption in the glass substrates and the alignment layers on the ITO films. It should also be noted that the simulation did not take into account the ITO absorption. Additionally, uneven alignment of the LC molecules on the glass substrates could have contributed to the difference between *n*_e_ and *n*_eff_ at zero voltage. The full width at half maximum (FWHM) of the simulated defect peaks is notably narrower than that of the experimental data. This discrepancy can be primarily ascribed to the instrumental resolution (≥2 nm) and may be due to inconsistencies in the direction of the LC molecules caused by interface roughness or other unpredictable factors, such as imperfect or defective fabrication of the dielectric multilayers.

**Figure 7:**
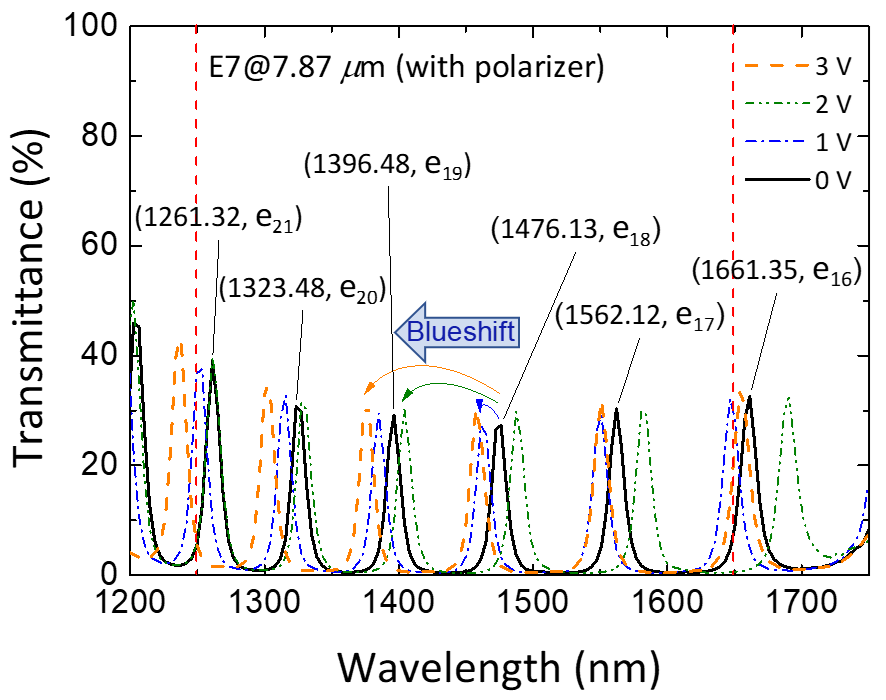
Polarized transmission spectra of the 7.87 μm thick PC/LC cell at *V* = 0 V_rms_, 1 V_rms_, 2 V_rms_, and 3 V_rms_.

Figure 7 displays the spectral profiles and variations in wavelength of the *e*-mode transmission peaks within the PBG structure at various applied voltages. The five *e*-mode signals at zero voltage can be identified and assigned as e_17_ (1562.12 nm), e_18_ (1476.13 nm), e_19_ (1396.48 nm), e_20_ (1323.48 nm), and e_21_ (1261.32 nm) in the wavelength range of 1250–1650 nm. To enable practical use in the devised spectrometric module and the like, it is necessary to shift a particular defect-mode peak from beyond 1650 nm to below 1650 nm by increasing *V*. Accordingly, the e_16_ peak at 1661.35 nm at *V* = 0 was identified for this purpose. As shown in Figure 7, while each *e*-mode peak presented a blueshift at *V* = 1 V_rms_, the shift extent was not significant. Taking the e_18_ mode as an example, when the voltage increased to 1 V_rms_, the peak exhibited a blueshift from 1476.13 nm to 1461.05 nm, indicating a wavelength shift of only 15.08 nm. When *V* increased to 2 V_rms_, the range of blueshift expanded, approaching the wavelength of the next higher order of the *e*-mode peak in the field-off transmission, as demonstrated by the essential blueshift of 72.02 nm for the e_18_ mode in response to the 2-V_rms_ voltage. It is worth mentioning that the significant blueshift observed at *V* = 2 V_rms_ can be attributed to the significant change in the LC director and, in turn, the effective refractive index compared with that at *V* = 1 V_rms_. The most noteworthy result was observed when *V* increased further to 3 V_rms_, where each *e*-mode peak could sweep across to the initial position of the next order of the defect mode. For instance, the e_18_ peak at *V* = 3 V_rms_ could blueshift and “exceed” the wavelength of the e_19_ peak at null applied voltage. These results affirmed that the entire spectral range of interest can be covered by increasing the cell thickness from 5.15 μm to 7.87 μm. Moreover, the experimental data show that the PC/LC cell of *d* = 7.87 μm required less than *V* = 3 V_rms_ to scan the entire wavelength range of 1250–1650 nm, greatly reducing the operating voltage to align with the promotion of environmental sustainability. These results are highly satisfactory, manifesting the potential for practical applications.

**Figure 8:**
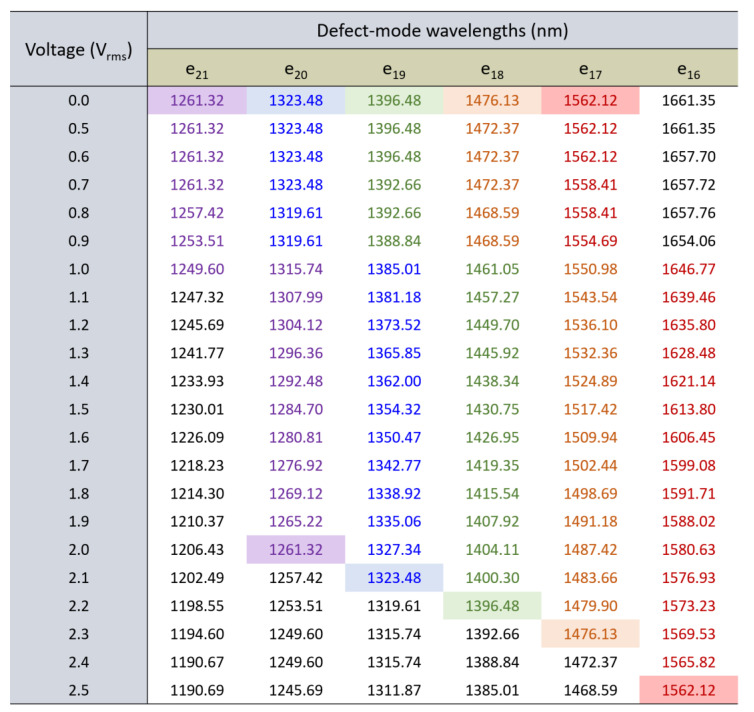
Spectral tunability of the *e*-mode peaks (e_16_ to e_21_) under various amplitudes of an electrical stimulus. The applied voltage is increased from *V* = 0 to 2.5 V_rms_ by a 0.1-V_rms_ increment.

To investigate the tunability for spectral scanning under various voltages, we collected *V*-dependent wavelength profiles and retrieved the wavelength data as shown in Figure 8. It is important to note that a chart-like figure, instead of a typical table, is presented here. A chart ensures that the wavelengths discerned or highlighted by their corresponding colorful backgrounds are more visually represented in a clear and comprehensive manner. This helps illustrate how an *e*-mode order “eventually” blueshifts to the wavelength of the next higher order at successively increasing voltages. The chart showcases the detailed spectral characteristics of wavelength tunability of defect modes based on the electrically controlled birefringence effect as *V* ramped up from 0 to 2.5 V_rms_. For example, the e_21_ peak required only 1 V_rms_ to scan the spectral span from 1261.32 to 1249.60 nm, which is very close to the lower limit of the 1250 nm boundary. Similarly, the e_20_ spectral peak scanned from 1323.48 to 1261.32 nm as *V* rose from 0 to 2 V_rms_. Other peaks (e.g., e_19_, e_18_, and e_17_) also exhibited blueshifts, blueshifting across their respective wavelength ranges under various external voltages. It is worth noting that the e_16_ defect-mode peak moved from a wavelength beyond 1650 nm to become inferior to the upper limit of the 1650 nm boundary when *V* increased from 0 to 1 V_rms_. In addition, when *V* increased further from 1 to 2.5 V_rms_, the e_16_ peak scanned through the desired spectral range from 1646.77 nm to 1562.12 nm, exactly the e_17_ wavelength at zero voltage. Based on the above demonstration and explanation, our study clearly shows the tunability in the refractive index of a field-responsive birefringent material that can be exploited for wavelength scanning for application in a new type of spectrometer design.

## 4. Conclusions

In conclusion, we demonstrated a tunable multichannel component as an active device that is capable of scanning the NIR spectrum of *λ* = 1250–1650 nm based on the electrically controlled birefringence effect in a homogeneously aligned LC cell. Our proposed PC/LC microcavity was fabricated by sandwiching nematic LC as a defect layer between two dielectric multilayers whose PBG covers the NIR range of interest. Inserted between the cell and a light source was an NIR linear polarizer, allowing the normally incident beam to be linearly polarized and the defect modes associated with *e*-waves. We started by investigating birefringence and dispersive behaviors of the nematic LC E7 in the NIR region using an Abbe refractometer and the extended Cauchy equations to grasp the wavelength-varying refractive indices. We simulated the polarized transmission spectra of PC/LC cells of various cell thicknesses. The experimental and simulated defect modes were assigned to their *e*-mode orders using Equation (3). Tunability in wavelength of the defect modes was achieved by means of lowering *n*_eff_ from *n*_e_ to *n*_o_ induced by increasing applied voltage, thereby provoking the blueshifts of the defect-mode peaks. By employing ball spacers of 8 μm in diameter to determine the thickened gap of the E7-filled cavity, we successfully realized a scanning multichannel component characterized by the low operating voltage of merely 2.5 V_rms_. The electro-optical properties and tunable spectral features of the PC/LC photonic structure reported in this work illustrate the potential for a wide range of applications in various tunable NIR components and devices, including miniature monochromators, spectrometers, multichannel filters for optical communications, and energy-saving waveguides, highlighting the significance of this study for the field of optical engineering.

## Figures and Tables

**Table 1:** Spectral features of defect modes through simulation for a range of PC/LC cell thicknesses in the field-off state. The figure in brackets labels the order of an *e*-mode feature.

Cell Gap (μm)	Defect-Mode Wavelengths (1250 nm < *λ* < 1650 nm) and the Order Numbers						
	Peak 1 (nm)	Peak 2 (nm)	Peak 3 (nm)	Peak 4 (nm)	Peak 5 (nm)	Peak 6 (nm)	Number of Peaks
9.0	1265 [24]	1321 [23]	1382 [22]	1449 [21]	1523 [20]	1605 [19]	6
8.0	1303 [21]	1369 [20]	1442 [19]	1524 [18]	1616 [17]		5
7.0	1281 [19]	1353 [18]	1435 [17]	1527 [16]	1631 [15]		5
6.0	1254 [17]	1334 [16]	1429 [15]	1530 [14]			4
5.0	1309 [13]	1413 [12]	1534 [11]				3
4.6	1316 [12]	1430 [11]	1565 [10]				3
4.0	1273 [11]	1395 [10]	1540 [9]				3
3.0	1369 [7]	1548 [6]					2

**Table 2:** Calculated defect-mode shifts in wavelength at various voltages. The blueshift of each *e*-mode varies from 0 at 0 V to a nonzero value at a voltage *V* given in the left column. (*d* = 5.15 μm).

Voltage (V_rms_)	Defect-Mode Wavelength Shifts (nm)			
	e_13_	e_12_	e_11_	e_10_
0	0	0	0	0
0.5	0.47	0.53	1.04	1.35
1	2.69	3.11	4.08	4.64
1.5	21.87	25.01	27.38	29.51
2	42.39	47.78	54.59	57.06
3	68.96	77.08	82.02	100.38
6	85.74	95.12	104.43	116.66
9	90.23	103.21	108.92	124.02
15	94.47	109.43	113.29	125.11
25	97.39	111.66	118.18	128.99
40	98.73	115.72	123.05	139.19

## Data Availability

The authors confirm that the data supporting the findings of this study are available within the article.
